# A statistically compiled test battery for feasible evaluation of knee function after rupture of the Anterior Cruciate Ligament – derived from long-term follow-up data

**DOI:** 10.1371/journal.pone.0176247

**Published:** 2017-05-01

**Authors:** Lina Schelin, Eva Tengman, Patrik Ryden, Charlotte Häger

**Affiliations:** 1 Department of Community Medicine and Rehabilitation, Physiotherapy, Umeå University, Umeå, Sweden; 2 Department of Statistics, Umeå School of Business and Economics, Umeå University, Umeå, Sweden; 3 Department of Mathematics and Mathematical Statistics, Umeå University, Umeå Sweden; University of the Sciences in Philadelphia, UNITED STATES

## Abstract

**Purpose:**

Clinical test batteries for evaluation of knee function after injury to the Anterior Cruciate Ligament (ACL) should be valid and feasible, while reliably capturing the outcome of rehabilitation. There is currently a lack of consensus as to which of the many available assessment tools for knee function that should be included. The present aim was to use a statistical approach to investigate the contribution of frequently used tests to avoid redundancy, and filter them down to a proposed comprehensive and yet feasible test battery for long-term evaluation after ACL injury.

**Methods:**

In total 48 outcome variables related to knee function, all potentially relevant for a long-term follow-up, were included from a cross-sectional study where 70 ACL-injured (17–28 years post injury) individuals were compared to 33 controls. Cluster analysis and logistic regression were used to group variables and identify an optimal test battery, from which a summarized estimator of knee function representing various functional aspects was derived.

**Results:**

As expected, several variables were strongly correlated, and the variables also fell into logical clusters with higher within-correlation (max ρ = 0.61) than between clusters (max ρ = 0.19). An extracted test battery with just four variables assessing one-leg balance, isokinetic knee extension strength and hop performance (one-leg hop, side hop) were mathematically combined to an estimator of knee function, which acceptably classified ACL-injured individuals and controls. This estimator, derived from objective measures, correlated significantly with self-reported function, e.g. Lysholm score (ρ = 0.66; p<0.001).

**Conclusions:**

The proposed test battery, based on a solid statistical approach, includes assessments which are all clinically feasible, while also covering complementary aspects of knee function. Similar test batteries could be determined for earlier phases of ACL rehabilitation or to enable longitudinal monitoring. Such developments, established on a well-grounded consensus of measurements, would facilitate comparisons of studies and enable evidence-based rehabilitation.

## Introduction

Rupture of the anterior cruciate ligament (ACL) is a common injury especially in individuals who participate in sports [1, 2]. Treatment involves either physiotherapy in combination with reconstructive surgery, or physiotherapy alone. Regardless of treatment, individuals still often suffer from varying extents of impaired knee function, both in the short [3, 4] and long-term perspective despite completing rehabilitation [5–8]. Such reduced knee function may be manifested by, for instance, instability, pain, swelling, decreased range of motion, joint stiffness, reduced physical capacity or decreased activity level in everyday tasks, but particularly with regard to sports and recreational activities. Consequently, attempts to determine knee function often combine several assessment tools covering different aspects of knee function based mainly on clinical examination, knee-specific scores and functional tests. The latter are aimed at capturing indicators of physical capacity, e.g. muscular strength, balance, motor coordination etc. There is, however, still no consensus on which outcome measures to use, which makes comparisons across studies difficult and leads to a lack of evidence for specific interventions. In the clinic, self-reported questionnaires and examiner-administrated knee scores such as the International Knee Documentation Committee 2000 subjective form (IKDC) [9], Knee injury and Osteoarthritis Outcome Score (KOOS) [10] or Lysholm questionnaire [11] are commonly used, and often in combination with a strength measurement and a hop task. Regarding functional assessments, different test batteries have been suggested [12–14]. A *test battery* in this context refers to a set of functional tests. A test battery consisting of three commonly used hop tests (vertical hop, one-leg hop for distance, and side hop), has shown a high ability to discriminate between the injured and non-injured leg of individuals with ACL injury [12]. Another test battery consisting of four hop tests (one-leg hop for distance, 6-m timed hop, triple hop for distance and crossover hop for distance) has also been demonstrated to be reliable and valid [14, 15]. Yet another test battery, consisting of knee-extension, knee-flexion and leg-press tests, discriminates between strength of the injured and the non-injured leg [13]. The full potential of such test batteries is not always achieved, since the specific test results are most often evaluated separately. The statistical methodology for a research question related to a single outcome variable is often straightforward. Typically two (or more) groups are compared with respect to a single variable using a statistical test [e.g. [5, 6, 8]]. Such tests are sometimes suitable to answer research questions, but single outcome variable analysis might not reveal all of the information contained in the data. It is, for example, possible to find significant differences between two groups when studying two variables simultaneously, while a separate analysis for each of them would not reveal any significant group differences. Hence, it would be desirable to analyze several variables simultaneously.

**Fig 1:**
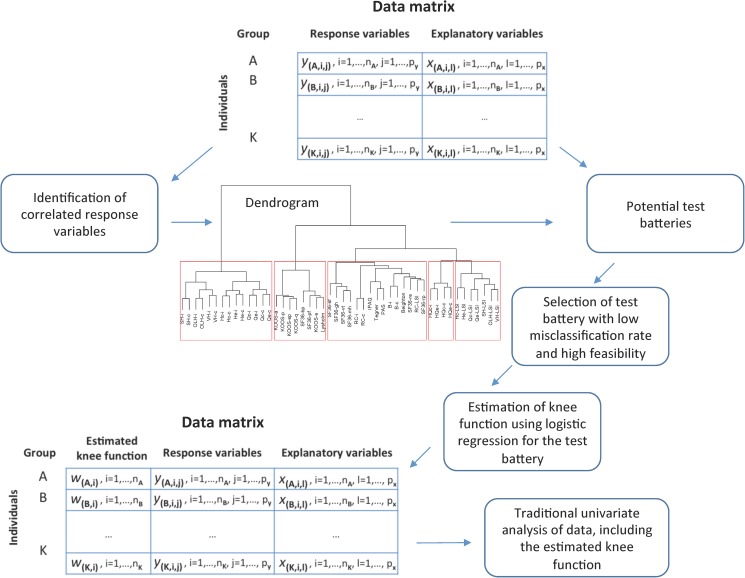
Illustration of the data structure and the statistical approach. First, correlation analysis combined with cluster analysis is applied to better understand the relationship between all outcome variables. Potential test batteries are then investigated using logistic regression and subsequently evaluated based on their misclassification rate and on their feasibility. The combined outcomes of the final test battery result in an estimator of knee function, again using logistic regression. Finally, this new variable (estimator of knee function) is analyzed using traditional statistical approaches such as Spearman rank correlation and Wilcoxon rank sum test.

Correlation analysis and cluster analysis can be used to understand relationships between variables. Examples of statistical methods for dimension reduction are factor analysis, principal component analysis (PCA) and logistic regression. Such methods may be used to combine information from several tests into a more valid estimator of knee function. However, inclusion of many variables in one model may make interpretation difficult. Alternatively, building a model based on a selected subset of variables may result in a model that is easier to interpret. This could be achieved by applying a statistical approach that can determine which knee tests that would be necessary and which would be redundant. In the present paper, a statistical approach was implemented to define a comprehensive and feasible test battery (Fig 1) that would be more discriminative than each of the included single subtests applied separately. To the best of our knowledge, such an approach has not been attempted with regard to knee function assessment.

We have utilized data from our long-term follow-up to implement the proposed statistical approach and suggest a test battery to evaluate long-term knee function after ACL injury. The statistical approach used to identify potential test batteries is based on logistic regression models. The models in question should be able to discriminate between different knee function abilities. This could be quantified by considering the models *misclassification rate*; defined as the proportion of incorrectly classified individuals, with and without ACL injury, when using the model. A low misclassification rate implies in our case, that the test battery better discriminates between injured individuals and healthy-knee controls. Since there may be a bilateral decrease of knee function following a unilateral injury [16–18], test batteries evaluated in both injured individuals and healthy-knee controls may provide additional information about which functional tasks to include in the test battery.

Due to the difficulties in defining optimal knee function from a rehabilitation perspective, it would be advantageous to measure all potential aspects of knee function. However, for practical reasons the test battery often needs to be relatively small and *feasible* (*i*.*e*. running the test should be relatively quick and not require extraordinary/specialized equipment). This aspect is also considered when compiling the proposed test battery. Finally, the variables in the final test battery are combined to an estimator of knee function using logistic regression.

In addition to having a model that facilitates an estimation of the overall knee function, it also seems highly important to understand the relationship between all outcome variables, e.g. to identify groups of variables that are correlated to each other. This problem may be addressed by using correlation analysis combined with cluster analysis, as we present later. Highly correlated variables might contribute with similar information, *i*.*e*. little information is lost if a group of strongly correlated variables is represented by just one of these variables.

Further, in the case of knee function, previous studies have found low or moderate correlations between patient-reported outcome scores and variables from functional tests [19–22]. This suggests that single functional tests are generally not able to measure overall knee function. A compiled index derived from a representative test battery would also be more likely to correspond with self-reported function.

The aim of this paper was to investigate the possibility of applying such a statistical approach to a large set of knee assessments, thereby detecting highly correlated variables and filter them down to a suggestion of a comprehensive and yet feasible clinical test battery consisting of only a few tests to be used in long-term evaluation after ACL injury. If proven useful, the suggested method could be applied to propose test batteries appropriate for acute and sub-acute phases of ACL rehabilitation, as well as monitoring and evaluation of other disorders in many clinical fields.

## Method

### Participants

The KACL20-study (Knee injury—Anterior Cruciate Ligament after more than 20 years [7, 23]) is a long-term follow-up with a cross-sectional design, where 70 individuals who had suffered unilateral ACL injury, on average 23 (17–28) years ago, were compared to 33 healthy-knee controls matched for age and sex. Basic individual characteristics are found in Table 1, and detailed outcome aspects related to physical activity, hop performance, and knee strength have been reported elsewhere [7, 23]. All participants were presented with written and oral information about the study and gave their written informed consent according to the declaration of Helsinki. The project was approved by the Regional Ethical Review Board in Umeå, Sweden (Dnr. 07-155M and Dnr. 08-211M).

**Table 1:** Participant characteristics.

	Individuals with ACL injury	Healthy-knee controls
Number of participants	70	33
Surgery/non-surgery	33/37	N/A
Men/women	44/26	21/12
Age at follow-up test	46.9 (5.4)	46.7 (5.0)
BMI	28.1 (4.1)	24.6 (2.5)

The characteristics presented as number of individuals or mean (standard deviation).

### Outcome variables

The variables were obtained from a large set of knee tests, questionnaires and scores considered to have good measurement properties, which are commonly used in research and clinics for evaluation of ACL rehabilitation [12, 24]: we chose nine *functional tests* (including hop tests, strength measurements and balance tests), four *self-reported questionnaires*, and three *examiner-administrated scores*, resulting in a total of 48 outcome variables. Brief descriptions of all 48 variables and information about their feasibility are found in Table 2. The different hop tasks and the one-leg balance have been comprehensively described in earlier papers [7, 25]. The variables obtained from functional tests were recorded in a movement laboratory; U-motion lab Umeå University. Participants performed the one-leg hop for distance (OLH), one-leg vertical hop (VH), rise from chair (RC), side hop (SH) and one-leg balance (B) on both the injured (i) and the non-injured (c) leg. For healthy-knee controls both the non-dominant leg (i) and the dominant leg (c) were included. For each exercise both absolute measurements (e.g. maximal hop distance on each leg) and relative measurements, such as the Limb Symmetry Index (LSI), were considered. The strength variables were obtained from peak isokinetic measurements where knee flexion torque (representing hamstrings, H) and knee extension torque (representing quadriceps, Q) in concentric and eccentric contractions were measured on both legs (for details see Tengman et al. b 2014 [23]). All strength variables were quantified in relation to the body weight (Nm/kg) of the individual. LSI and the ratio between hamstrings and quadriceps peak torque (H:Q ratio) were also calculated.

**Table 2:** A brief description of the 48 outcome variables included in the analysis.

Variable			Short description	P-value	T:E- index
**Functional tests (hops and balance)**					
	One-leg hop		Absolute hop length in m		1:1
		*OLH-i*	*Non-dominant/injured leg*	0.820	
		*OLH-c*	*Dominant/non-injured leg*	0.143	
		*OLH-LSI*	*Limb symmetry index*	0.001^+^	
	Vertical hop		Absolute hop height in cm		1:1
		*VH-i*	*Non-dominant/injured leg*	0.106	
		*VH-c*	*Dominant/non-injured leg*	0.632	
		*VH-LSI*	*Limb symmetry index*	0.105	
	Rise from chair		Number of rises from a chair		1:1
		*RC-i*	*Non-dominant/injured leg*	0.028^+^	
		*RC-c*	*Dominant/non-injured leg*	0.012^+^	
		*RC-LSI*	*Limb symmetry index*	0.690	
	Side hop		Number of side hops		1:1
		*SH-i*	*Non-dominant/injured leg*	0.000^+^	
		*SH-c*	*Dominant/non-injured leg*	0.004^+^	
		*SH-LSI*	*Limb symmetry index*	0.004^+^	
	One-leg balance		Number of floor-supports		1:1
		*B-i*	*Non-dominant/injured leg*	0.022^*-*^	
		*B-c*	*Dominant/non-injured leg*	0.000^-^	
**Functional tests (strength measurements)**					
	Quadriceps		Concentric strength		3:2
		*Qc-i*	*Non-dominant/injured leg*	0.000^+^	
		*Qc-c*	*Dominant/non-injured leg*	0.132	
		*Qc-LSI*	*Limb symmetry index*	0.005^+^	
			Eccentric strength		3:2
		*Qe-i*	*Non-dominant/injured leg*	0.002^+^	
		*Qe-c*	*Dominant/non-injured leg*	0.179	
		*Qe-LSI*	*Limb symmetry index*	0.027^+^	
	Hamstrings		Concentric strength		3:2
		*Hc-i*	*Non-dominant/injured leg*	0.076	
		*Hc-c*	*Dominant/non-injured leg*	0.159	
		*HC-LSI*	*Limb symmetry index*	0.401	
			Eccentric strength		3:2
		*He-i*	*Non-dominant/injured leg*	0.259	
		*He-c*	*Dominant/non-injured leg*	0.536	
		*He-LSI*	*Limb symmetry index*	0.255	
	Strength ratios		Ratio between concentric (or eccentric) hamstrings and quadriceps strength		3:2
		*HQc-i*	*Non-dominant/injured leg*	0.136	
		*HQc-c*	*Dominant/non-injured leg*	0.837	
		*HQe-i*	*Non-dominant/injured leg*	0.054	
		*HQe-c*	*Dominant/non-injured leg*	0.591	
**Examiner-administrated scores**					
	Beighton		A score measuring hypermobility	0.020^-^	1:1
	Tegner		Tegner activity scale measuring knee specific physical activity	0.000^+^	1:1
	Lysholm		Knee function score	0.000^+^	1:1
**Self-reported questionnaires**					
	KOOS		Knee injury and Osteoarthritis Outcome Score		2:1
		*KOOS-s*	*Symptoms*	0.000^+^	
		*KOOS-p*	*Pain*	0.000^+^	
		*KOOS-a*	*Function in activities of daily living*	0.000^+^	
		*KOOS-sp*	*Sports and recreation*	0.000^+^	
		*KOOS-q*	*Knee-related quality of life*	0.000^+^	
	PAS		Physical Activity Scale	0.044^+^	1:1
	IPAQ		International Physical Activity Questionnaire	0.247	1:1
	SF-36		Patient-reported survey of patient health		2:1
		*SF36-pf*	*Physical functioning*	0.000^+^	
		*SF36-rp*	*Role physical*	0.043^+^	
		*SF36-bp*	*Bodily pain*	0.000^+^	
		*SF36-gh*	*General health*	0.005^+^	
		*SF36-vt*	*Vitality*	0.151	
		*SF36-sf*	*Social functioning*	0.646	
		*SF36-re*	*Role emotional*	0.353	
		*SF36-mh*	*Mental health*	0.317	

The P-value column states if there is a significant difference or not between individuals with ACL injury and controls, controlling for age and sex. If a significant difference is present, the symbol + indicates that the healthy-knee controls have a higher value than the ACL group while the symbol—indicates the opposite. T:E-index indicates how demanding the variable is to collect considering time (T, score 1–3) and equipment (E, score 1–2). Higher values correspond to lower feasibility.

All individuals answered several knee-specific and more general questionnaires including: KOOS [10], Physical Activity Scale (PAS) [12, 26], International Physical Activity Questionnaire (IPAQ), 36-Item Short Form Health Survey (SF 36) [27]. For KOOS and SF-36 each sub score was considered as one variable. Lysholm score, Tegner activity scale, [11] and Beighton score were examiner administrated. See Table 2 for the complete list of all variables. In addition to the variables described above, some background variables were observed, including age, sex, and clinical history (*i*.*e*. ACL-injured or healthy-knee control).

### Feasibility index

A clinical test battery should be feasible. We therefore asked ten expert physiotherapists to independently rank all included functional assessments and questionnaires according to time requirement and to which extent specific equipment is needed. The ranking from the physiotherapists is presented in Table 2 as a T:E-index, where T stands for time and E for equipment. Regarding time, a ranking of 1, 2 or 3 corresponds to less than 15 minutes, 15–30 minutes or more than 30 minutes respectively. The estimated time demand includes the needed time for preparation, execution and data registration.

Regarding equipment, a ranking of 1 corresponds to basic equipment always being available, while a ranking of 2 implies advanced equipment or licenses. The T:E-indexes in Table 2 were obtained as the median of the answers given by the physiotherapists. The aim with the T:E-index was to allow comparisons between test batteries regarding feasibility.

### Statistical analysis

In order to better understand the relationship between all outcome variables we use correlation analysis combined with cluster analysis, and to statistically derive potential test batteries we use logistic regression models. To evaluate the models, *i*.*e*. the test batteries; we consider each models misclassification rate. The statistical analysis is summarized in Fig 1 and the methodology details are presented below.

Correlation analysis combined with hierarchical cluster analysis was used to identify highly correlated variables. First, the Spearman’s rank correlation was used to calculate the correlation between all pairs of variables, denoted *ρ*_*ij*_, where *i* and *j* are indexes for the variables. Next, a dendrogram (*i*.*e*. tree describing the relative distance between the variables) was obtained using hierarchical cluster analysis [28] with Ward linkage and a distance matrix *D* for which the elements were: one minus the absolute correlation, *i.e., d*_*ij*_ = 1 − |*ρ*_*ij*_|. The cluster analysis resulted in a dendrogram where highly correlated variables were grouped in clusters. Each cluster corresponds to one branch of the dendrogram.

Test batteries were obtained by selecting 1–30 variables from the functional assessments defined in Table 2. Here, different selection strategies were considered. For small batteries (1–4 variables) all possible combinations were investigated, and for all larger batteries (5–20 variables) 10,000 randomly sampled sets of variables were considered. In addition, we also considered the complete test battery when including all 30 functional test variables.

For each test battery logistic regression was used to model knee function as a function of the variables in the respective test battery. This was done as follows. Let *Y* denote the binary variable reflecting the clinical history of the patient, that is; 1 for healthy controls, and 0 for ACL-injured. Let (*X*_*b*1_,…,*X*_*bk*_) denote the variables used in the *b*th battery. Logistic regression [29], using the above explanatory variables, but no interaction terms, was used to model the probability *w*_*b*_ that the patient is healthy, by

$$\text{logit}({\text{w}}_{\text{b}})=\beta_{b0}+\beta_{b1}\;{\text{x}}_{b1}+\cdots +\beta_{bk}\;{\text{x}}_{bk}.$$

Note that *w*_*b*_ may be interpreted as an estimator of the individuals’ relative knee function, where 0 is bad and 1 is good. The coefficient *β*_*bi*_ should be interpreted in the following way: a one-unit increase in the variable *x*_*bi*_, holding all other variables at fixed values, corresponds to a 100 exp(*β*_*bi*_)% increase in the odds of being a healthy control.

Each battery was evaluated by considering the corresponding model’s misclassification rate and how feasible the included variables are in the clinic (see below). The commonly used misclassification rate was estimated using leave-one-out cross validation, and should be interpreted as the probability of being classified into the wrong group. The models could include both significant and insignificant variables.

The correlation between the estimated knee function w from the final test battery and other variables were calculated using Spearman’s rank correlation and, for two group comparisons, Wilcoxon’s rank sum test was used. All statistical analyses were performed using the software R, version 2.15.2.

### Compilation of test battery

Altogether about 72 000 test batteries, representing different combinations of the included test variables, were selected and evaluated. All test batteries with a misclassification rate lower than 0.2 were investigated further. This cut-off value was chosen arbitrarily to define a subset of reasonable size for further investigation. The feasibility of those test batteries was estimated by the sum of the variables’ feasibility indexes. For example, a battery including the variable OLH-i from the one-leg hop for distance test and the variable Qc-i from the concentric contraction representing quadriceps has an aggregated feasibility index of 7 according to Table 2. A battery with a low index is regarded as highly feasible. Further, if a test battery includes the variable OLH-i, the variable OLH-c will be available without increasing the feasibility. For the identified functional tests, we considered all possible combinations of variables available for these tests. A condition for the final test battery, based on clinical relevance, was that it would mainly consist of variables related to the injured leg. A relevant final test battery was compiled using these established criteria, and in combination with existing clinical evidence.

## Results

**Fig 2:**
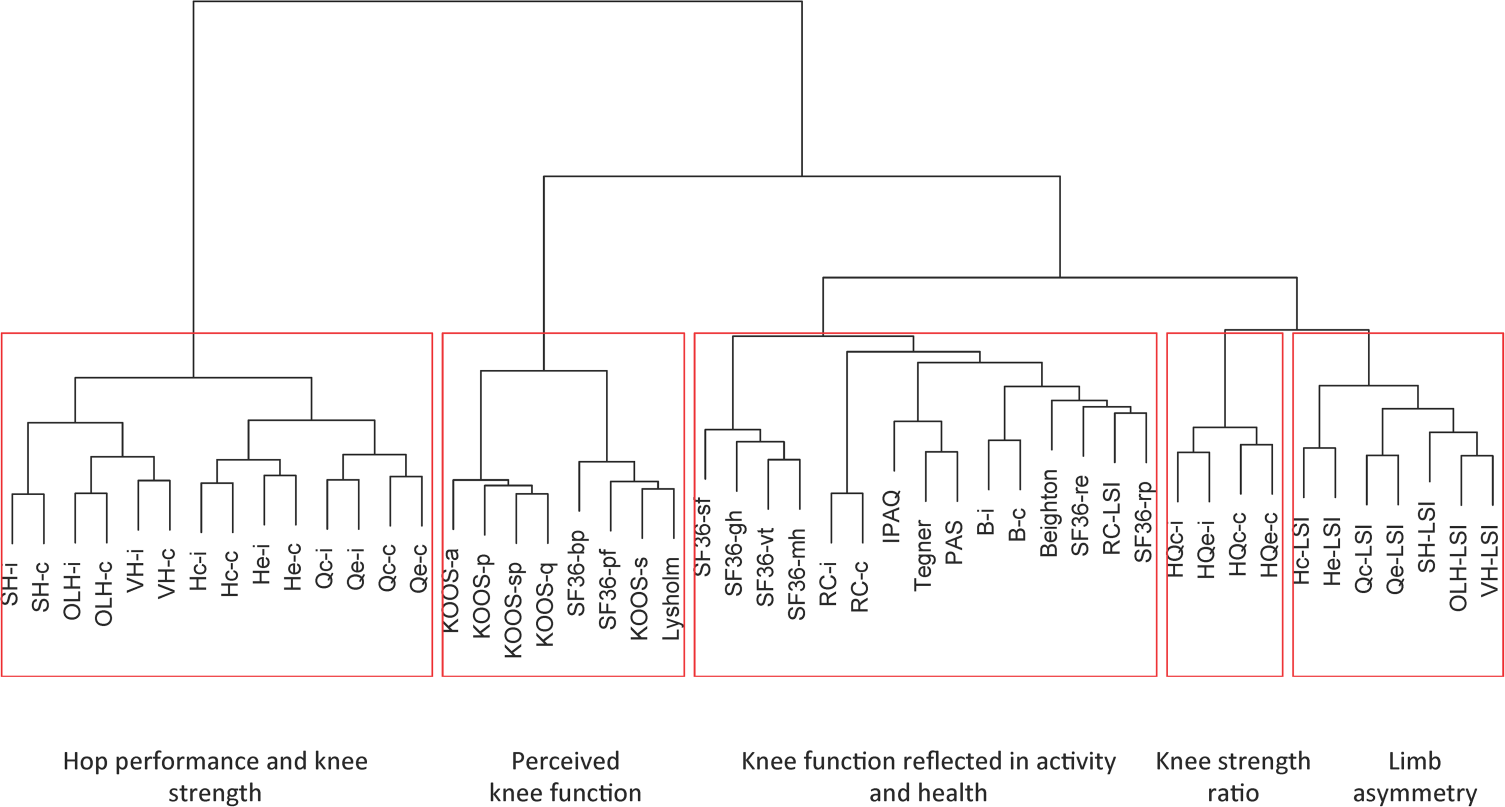
Results from Ward hierarchical cluster analysis based on Spearman correlation. The analysis resulted in five clusters: the *Hop performance and knee strength* cluster is associated with absolute measurements of functional tests and knee strength measures; the *Perceived knee function* cluster is linked with scores and questionnaires; the *Knee function reflected in activity and health cluster* contains a mixture of variables of different character; the *Knee strength ratio* and the *Limb asymmetry* clusters were mainly associated with relative measurements between legs (LSI) in functional tests.

The data from the KACL20-study used in the present paper included data from both healthy-knee controls and ACL-injured individuals. When applying correlation analysis combined with hierarchical cluster analysis the variables fell into five major clusters that in fact represented clinically meaningful dimensions of knee functions. Generally, the pairwise correlation within the clusters was significantly higher than between the clusters (p-value = 0.005), see Fig 2. Each cluster broadly represents different dimensions of knee function; Cluster I: *the Hop performance and knee strength* included all absolute variables from the functional tests with the exception of the variables from RC and B. Cluster II: *the Perceived knee function* included most of the *self-reported* questionnaires and *examiner-administrated scores* related to perceived knee function, including the five sub scores of KOOS, Lysholm, SF36-bp and SF36-pf. Cluster III: *Knee function reflected in activity and health* was the most diverse group and included variables related to activity (Tegner, PAS) and general health (SF36), RC (RC-c, RC-i, RC-LSI), and B (B-c, B-i). Cluster IV: *the Knee strength ratio* and Cluster V: *the Limb asymmetry* were rather closely related and contained all the relative functional tests variables with the exception of RC-LSI. The average absolute correlations between variables within cluster I-V were 0.55 (SD = 0.11), 0.61 (SD = 0.15), 0.14 (SD = 0.13), 0.39 (SD = 0.08), and 0.29, (SD = 0.13) respectively. The average absolute correlation between variables from different clusters was 0.12 (SD = 0.05).

**Fig 3:**
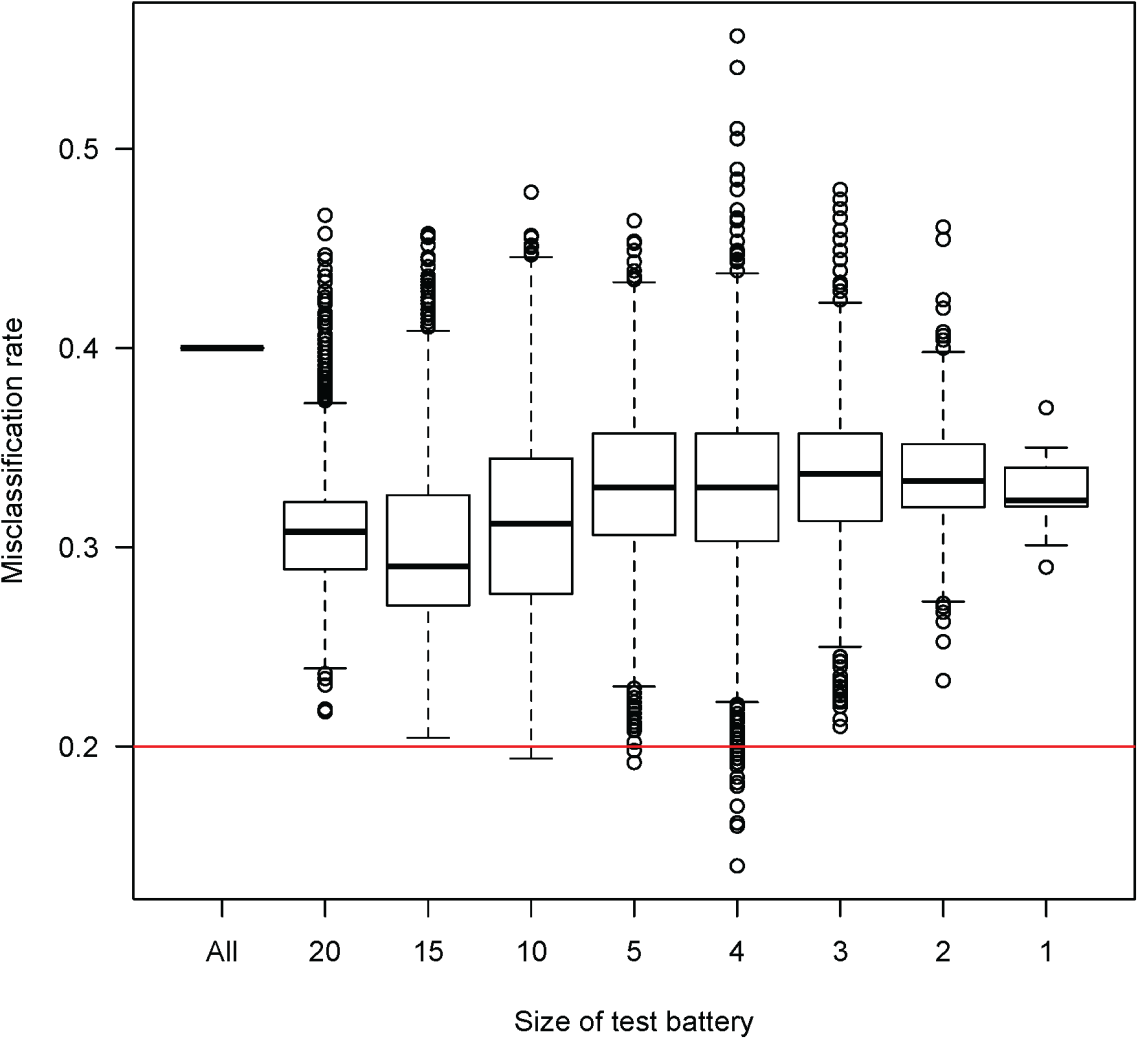
Misclassification rates for different sizes of test batteries. Misclassification rates for about 72 000 test batteries of different sizes, representing different combinations of the included test variables. The size of the test battery is the number of included variables. The misclassification rate should be as low as possible. The results for combinations consisting of 5, 10, 15 and 20 variables are based on 10000 random samples. The horizontal line indicates our threshold (0.2) for the highest acceptable misclassification rate.

Potential test batteries, including only variables from functional tests, were obtained by selecting 1–30 variables from the clusters. The misclassification rate, defined as the proportion of incorrectly classified individuals with and without ACL injury, when using all 30 strength and functional test variables, was 0.40. The median misclassification rates for test batteries with 1–20 variables varied between 0.29 (15 variables) and 0.36 (3 variables). Interestingly, the test batteries with the lowest (and also highest) misclassification rates were found among test batteries with 3–5 variables, suggesting that a battery with few variables may more accurately reflect knee function (Fig 3).

We identified the following tests as typically connected to a low misclassification rate; one-leg hop for distance (OLH), side hop (SH), one-leg balance (B), rise from chair (RC), and quadriceps concentric (Qc) and eccentric (Qe) knee strength. Therefore, the misclassification rates for **all** combinations of variables related to these functional tests were additionally investigated. Table 3 shows some of the most interesting test batteries and illustrates how the misclassification rate and the total feasibility-index (indicating time and equipment requirements) change when variables are added to the model.

**Table 3:** Misclassification rates and the T:E-index for a selected subset of test batteries.

Variables		Misclassification rate	Total T:E- index
**1. One-leg hop, Side hop, One-leg balance, Quadriceps concentric strength**			
1	Qc-i	0.29	5
2	Qc-i, OLH-i	0.28	7
3	Qc-i, OLH-i, B-c	0.26	9
**4**	**Qc-i, OLH-i, B-c, SH-i**	**0.17**	**11**
5	Qc-i, OLH-i, OLH-c, B-c, SH-i	0.16	11
6	Qc-i, OLH-i, OLH-c, B-c, SH-i, SH-c	0.15	11
7	Qc-i, Qc-c, OLH-i, OLH-c, B-c, SH-i, SH-c	0.16	11
**2. One-leg hop, Rise from chair, One-leg balance, Quadriceps concentric strength**			
1	Qc-i	0.29	5
2	Qc-i, OLH-i	0.28	7
3	Qc-i, OLH-i, B-c	0.26	9
4	Qc-i, OLH-i, OLH-c, B-c	0.19	9
5	Qc-i, OLH-i, OLH-c, B-c, RC-i	0.17	11
6	Qc-i, Qc-c, OLH-i, OLH-c, B-c, RC-i,	0.18	11
**3. One-leg hop, Rise from chair, One-leg balance, Quadriceps eccentric strength**			
1	OLH-LSI	0.30	2
2	OLH-LSI, B-c	0.27	4
3	OLH-LSI, B-c, Qe-i	0.24	9
4	OLH-i, OLH-LSI, B-c, Qe-i	0.22	9
5	OLH-i, OLH-LSI, B-c, Qe-i, RC-i	0.20	11
6	OLH-i, OLH-LSI, B-c, Qe-i, RC-i, RC-LSI	0.20	11
**4. One-leg hop, Side hop, One-leg balance, Quadriceps eccentric strength**			
1	SH-i	0.32	2
2	SH-i, B-c	0.27	4
3	SH-i, B-c, OLH-i	0.22	6
4	SH-i, B-c, OLH-I, Qe-i	0.20	11
5	SH-i, B-c, OLH-i, OLH-c, Qe-i	0.17	11
6	SH-i, B-c, OLH-i, OLH-c, Qe-i, Qe-c	0.17	11

For each of the four examples, the first column shows the test battery of size one corresponding to the lowest misclassification rate. The second column shows the misclassification rate and the total T:E-index when one variable was added to the starting model. Each of the following rows indicated that one additional variable was added. The final selected test battery is marked in bold in the shaded area.

The analyses resulted in several models with misclassification rates below 0.2 and feasibility indexes below or equal to 11, some of which are shown in Table 3. Among them we selected a test battery with the variables OLH-i, SH-i, B-c, and Qc-i obtained from the functional assessments OLH, SH, B, and Qc. The battery has only four variables and three of them are related to the injured leg. The feasibility index for the battery was 11 and the estimated misclassification rate was 0.17 (Table 3). Based on the KACL20-data, the suggested battery resulted in the following model for estimating the patients overall knee function:

logit(w)=−1.1−6.3∙OLH-i+0.2∙SH-i−3.4∙B-c+2.7∙Qc-i.

**Fig 4:**
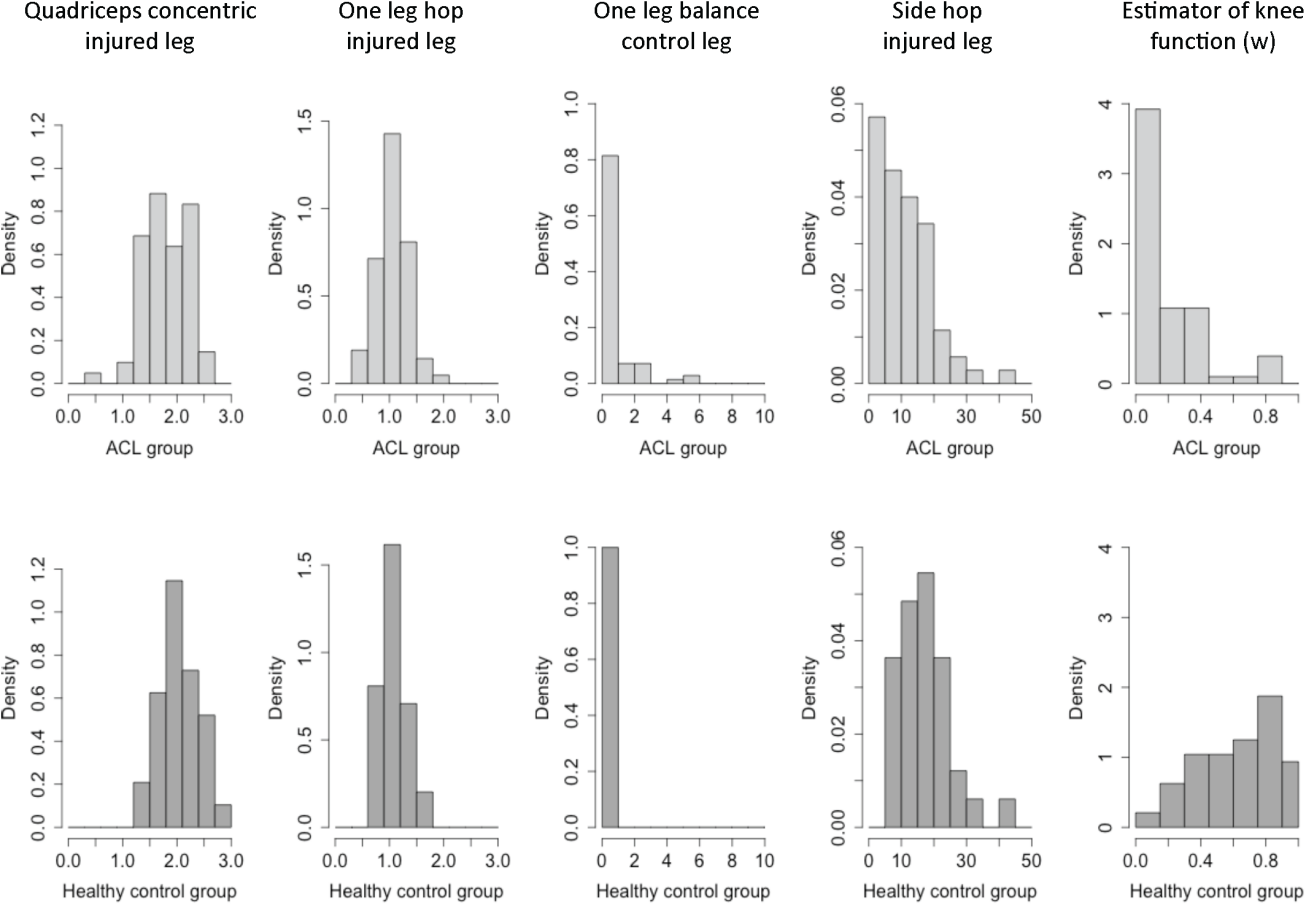
Distributions of the estimator of knee function and the test battery variables. The distribution of each of the variables included in the estimator of knee function (w) for each of the two groups, *i*.*e*. individuals with an ACL injury and healthy-knee controls. For the estimator of knee function, values close to 1 indicate a good knee function, and values close to 0 indicate the opposite. Quadriceps concentric strength was measured in Nm/kg; the one-leg hop for distance in meters, the one-leg balance in number of floor support, and the side hop in number of side hops.

Interpret the new outcome variable, *w*, as an estimator of the individuals’ knee function where 0 represents very low function and 1 indicates very good knee function. The distributions of the variables in the battery were similar between the ACL-injured and the healthy-knee controls, while the corresponding distributions for the estimated knee function *w* were more distinct, see Fig 4.

Interestingly, the estimator *w* was positively correlated with perceived knee function taken from Lysholm score (*ρ* = 0.66, p-value < 0.001), all subscales of KOOS (*ρ* = 0.36–0.60, p-values < 0.001), Tegner activity scale (*ρ* = 0.26, p = 0.008), and three sub scores (pf, bp, and gh) of SF36 (*ρ* = 0.23–0.47, p-values < 0.017) and negatively correlated with SF36-mh (*ρ* = -0.22, p-value = 0.028). No significant difference between sexes was found (p-value = 0.6). Further, there was no correlation between w and age (*ρ* = -0.12, p-value = 0.25).

## Discussion

The aim of this paper was to suggest a solid statistical selection process to derive a comprehensive and yet feasible clinical test battery with different functional aspects to be used in rehabilitation after ACL injury. The test battery may be used to characterize knee function following an ACL injury. In this specific case, for the purpose of suggesting a test battery for *long-term follow-up* after ACL injury, we used data from the KACL20-study to investigate which combination of variables that optimally distinguished ACL-injured and healthy-knee controls, while still being feasible and clinically relevant. We extracted a test battery with four variables related to functional tests that may be used as a complement to questionnaires and scores in the long-term perspective after injury. It is a true challenge to define “good knee function” and many dimensions need to be considered. Previously reported test batteries have used data without healthy-knee controls, *i*.*e*. they compared knee function between the injured and the non-injured leg [12, 14, 15]. However, several studies show that there might be decreased bilateral function after a unilateral ACL injury [16–18]. Therefore, it is essential that a test battery can also reliably discriminate between persons with an ACL injury and healthy-knee controls, since knee function may vary substantially across individuals, whether injured or not. This is accomplished by the suggested test battery, which distinguishes those with good knee function from those with less good knee function.

In the clinical setting and in research several different functional tests are used. Many of these test results are highly correlated, as corroborated in the present study (see Fig 2), and thus may provide similar information. Our study investigated which of the nine functional tests, and their related variables used in the KACL-study, that were able to optimally distinguish persons with bad knee function from those with good knee function. Even if adequate tests are used it is often difficult to interpret the combined information from several tests. In the construction of the new variable *w*, the statistical analysis identified two hop tasks from the same cluster, but from different branches (c.f. Fig 2). They are thus correlated, but represent different aspects of knee function. The OLH is an explosive maximal hop test for distance, which is performed in a forward direction and is assumed to be the most common test used in research and clinics after ACL injury [30, 31]. The SH on the other hand, is a multiple hop test, reflecting endurance as represented by a maximum number of hops which are performed in a medio-lateral direction. Thus, the OLH and the SH have different purposes and represent different coordinative function and performance. In addition, these tests challenge dynamic knee joint stability differently, where most likely the SH exerts higher demands on rotational stability (cf. [32, 33]) and may be more critical when evaluating capacity in the clinic [7]. For the SH, we have recently demonstrated that the test challenges knee stability substantially (data obtained from the same study population as in the present paper [32]). Both the OLH and SH tests are considered to have high reliability [12] and are considered highly relevant for evaluation of knee function after ACL injury [34].

In addition, the quadriceps concentric strength (Qc) and the one-leg balance test (B) were identified in the selected test battery. Regarding knee muscle strength, a review article by Palmieri-Smith et al. concludes that despite rehabilitation, knee muscle weakness is one of the main dysfunctions following ACL injury [35]. Isokinetic concentric quadriceps strength testing is frequently used in knee rehabilitation, is associated with self-reported knee function [36] and has high reliability [37]. Balance further adds yet another dimension, where studies indicate a reduced ability following ACL injury for the injured as well as the non-injured leg [16, 25]. The combination of the selected tests implies a range across various clinically relevant physical dimensions of knee function and thus seems appropriate.

In the present study, logistic regression is used to combine the information from the variables in the test battery, since the model, expressed in the contributing variables, is relatively easy to interpret. The results showed that the estimator of knee function, *w*, to a high extent discriminates individuals with ACL injury from healthy-knee controls. Other statistical approaches such as factor analysis or principal component analysis could alternatively be used to summarize information from several variables into a few factors or principal components. These factors or principal components may then be used in further analysis. However, our primary focus was to propose a test battery consisting of a few variables that are clinically observable and relevant. Since the different test batteries were evaluated using logistic regression, it was natural to use this approach to combine the available information. Once the dimension of the problem has been reduced, traditional statistical analyses for univariate data can be performed.

Our final choice of *w* was based on the calculations of misclassification rates, combined with expert reasoning regarding feasibility, where the latter is crucial for outcome measures to be used in the clinic. Interestingly, the misclassification rate typically decreased when variables were added to the model, up to models with five to six variables; then it increased again. The misclassification rate depends on the choice of cut-off for classification, here set to 0.5. It might not be the optimal cut-off, but it can still be used for comparing test batteries. The misclassification rate for the final test battery was 0.17, meaning that 17% of the individuals were misclassified using the chosen model. It would be possible to include additional variables related to the non-injured leg and thereby reduce the misclassification rate to 0.15, *i*.*e*., to classify two additional individuals correctly. Even though the T:E-index does not increase, a model with fewer variables is preferable due to interpretability. Moreover, calculation of the total T:E-index for a test battery was based on the assumption that a functional test is always performed on both legs, which is praxis in research and clinic, where the non-injured leg is used as a reference leg for comparisons. When data for a control group is available for comparisons, the performance of the non-injured leg may not be as necessary to observe. Our final estimator of knee function mainly included the variables discussed above that were related to the injured leg (i). However, for the one-leg balance test, the non-injured leg (c) is used. Indeed, as discussed above both the injured and non-injured leg display balance deficits after injury [16, 25].

The distributions for each of the variables included in *w* were similar within the two groups, as seen in Fig 4. This was not the case for the estimator of knee function where a clear difference in distribution was shown, demonstrating the capacity to discriminate between injured and healthy-knee controls. Nevertheless, some errors in misclassification could be expected considering the difficulty in clarifying objective criteria for who actually has good knee function, as is a common experience of clinical experts. A wide range of knee functions across individuals is expected, which may particularly be the case a long time after injury with increasing age and deconditioning; and also the case for non-injured individuals. In the KACL20 data set, which included individuals mainly in their forties, there were individuals that had been successfully rehabilitated and displayed knee function that was equally good as age-matched healthy-knee controls. Moreover, some of the controls showed results similar to injured individuals, and were therefore classified as injured. Indeed, as shown in Fig 3, a variation in knee function is present within both groups.

Lysholm and KOOS scores are well established and commonly used in ACL rehabilitation. Both scores have high reliability and validity [38, 39]. Our proposed estimator of physical knee function, w, correlated positively with both scores, indicating that it is concurrent with the individuals self-reported knee function. We also investigated the potential influence of age and sex, which are individual factors that have been shown to influence some of the outcome variables. For instance, Tegner activity level and KOOS differ between sexes and are negatively influenced by age [40–42]. Physical capacity, including balance, strength and hop ability is likewise reduced with increasing age and lower for women than for men [43, 44]. Similarly, our study did not show any correlation of estimated knee function with age, and no difference between sexes. This may depend on the fact that our material is based on a long-term follow-up of knee function after ACL injury and thus covers ages between 35 and 63 years. These older age groups might be more homogenous than the younger athletes mainly tested in the above-mentioned studies, although many of the individuals with ACL injury in our data were athletes prior to injury. The controls were matched for age and sex but, although strived for, there was no matching of physical activity level.

The tests were performed by cohorts in a unique long-term follow-up with comparatively extensive testing. However, for our aims this is a limitation, since a larger reduction in knee function might be expected shortly after an injury. Thus, the suggested test battery needs to be further validated in other cohorts. In addition, the statistical approach should be applied to data obtained from shorter-term follow-ups after injury to verify the usefulness of the proposed test battery for other stages of rehabilitation. The data used from the KACL20-study includes nine different common functional tests as well as established scores and questionnaires adopted in rehabilitation after ACL injury, and hence seem well suited for our aims. Even so, there are many other existing tests that could have been used, e.g. triple-jump, running-eight test. Recent research has also identified the lack of measures of quality of movement; such indicators could be crucial factors for the prediction of outcome in knee rehabilitation [30]. Kinematic and kinetic variables aimed at capturing movement quality during coordination tasks could provide such measures [45, 46] but are not so feasible in the clinic. Nevertheless, research using laboratory-based evaluation could be used to identify and validate the most important clinical outcome measures. For instance, Di Stasi et al. used kinematics and kinetic assessments during gait to validate a clinical test battery in relation to return-to-sport criteria [47]. Kinematically-derived variables may also be used to characterize how challenging different functional tests are with regard to dynamic knee stability and compare functions across groups of individuals [32, 48]. However, kinematic and kinetic analyses usually generate huge/large numbers of variables, and there is a need to reduce these into the most representative parameters. In this context the present statistical approach would be of particular value, especially in large data sets obtained from various functional tasks. Thus, the model may be used to identify appropriate variables rather then arbitrarily selecting them.

In addition to kinematic and kinetic recordings of functional tests, proprioception and laxity may influence knee function [49] and it would be desirable to include such measures to ensure that as many dimensions of physical knee function as possible were to be considered. Altogether, our proposed test battery is comprised of four different test variables, reasonably feasible and, when combined, proven to reliably discriminate knee function at least in the long term after injury to the knee. An advantage with this test battery compared to previously proposed ones [12–15], is that it includes both functional coordination tests and more direct knee muscle strength measurements. Further testing of the measurement properties (e.g., validity, reliability, sensitivity) of the suggested test battery in other long-term study populations, more than one year after ACL injury, is warranted.

## Conclusions

The present study shows that with a solid statistical approach, we were able to construct a comprehensive and yet feasible test battery for evaluation of knee function after ACL injury which is appropriate in the long-term perspective. Our estimator of knee function combined several aspects, and could be said to more coherently represent true knee function than a single variable is able to. Consensus regarding clinical functional test batteries for various stages of rehabilitation, along with a general health score and a knee-specific health score, would ensure evidence-based assessment of knee function in patients following an ACL injury and enable reliable monitoring of knee function throughout the different phases of rehabilitation. Further, it would make it possible to carry out powerful retrospective and prospective studies over longer timespans post-injury while facilitating comparisons across studies.
